# CRISPR-Cas immunity and mobile DNA: a new superfamily of DNA transposons encoding a Cas1 endonuclease

**DOI:** 10.1186/1759-8753-5-23

**Published:** 2014-08-26

**Authors:** Alison B Hickman, Fred Dyda

**Affiliations:** 1 Laboratory of Molecular Biology, National Institute of Diabetes and Digestive and Kidney Diseases, National Institutes of Health, Bethesda, MD 20892, USA

**Keywords:** DNA transposition, V(D)J recombination, CRISPR-Cas, Adaptive immunity, Casposon, Transposable elements, Cas1 nuclease protein

## Abstract

Mobile genetic elements such as DNA transposons are a feature of most genomes. The existence of novel DNA transposons can be inferred when whole genome sequencing reveals the presence of hallmarks of mobile elements such as terminal inverted repeats (TIRs) flanked by target site duplications (TSDs). A recent report describes a new superfamily of DNA transposons in the genomes of a few bacteria and archaea that possess TIRs and TSDs, and encode several conserved genes including a *cas1* endonuclease gene, previously associated only with CRISPR-Cas adaptive immune systems. The data strongly suggests that these elements, designated ‘casposons’, are likely to be *bona fide* DNA transposons and that their Cas1 nucleases act as transposases and are possibly still active.

## Background

Mobile genetic elements can modify the genomes of the organisms that harbor them, and their mobility is believed to be an important factor in evolution (reviewed in [1-5]). Mobile elements can affect their host by disrupting genes, modifying control regions, and by introducing new proteins or protein domains into novel genomic locations. One of the best known examples is the RAG1 protein of jawed vertebrates which is a key protein required for the functioning of the adaptive immune system [6], and whose catalytic domain originated from the transposase associated with *Transib* transposons [7].

One of the most exciting recent advances in microbiology has been the discovery that an adaptive immune system also exists in many bacteria and archaea (reviewed in [8-11]). CRISPR-Cas systems provide a mechanism for prokaryotes to incorporate short stretches of foreign DNA (‘spacers’) into their genomes to archive sequence information on ‘non-self’ DNA they have encountered, such as that of viruses or plasmids. This is called the adaptation stage of the immune process. Once integrated, these spacers serve as templates for the synthesis of RNA which then directs Cas nucleases to specific foreign nucleic acids in order to degrade them. Several different types of CRISPR systems have been identified, and each is associated with a distinct set of Cas proteins. Only two proteins, Cas1 and Cas2, appear to be strictly conserved among the various CRISPR systems, and they are both metal-dependent nucleases. The structure of the Cas1-Cas2 complex from *E. coli* strain MG1655 has been determined [12].

A recent report by Krupovic *et al.*[13] presents data suggesting that Cas1 proteins of CRISPR systems originated from a newly identified superfamily of DNA transposons that the authors call ‘casposons’. If true, an elegant symmetry emerges in the evolutionary history of the establishment of adaptive immune systems in higher eukaryotes and in bacteria and archaea. Furthermore, the discovery of a novel family of DNA transposases would be a significant addition to the known repertoire of mechanisms by which mobile elements are moved [14].

## Main text

**Figure 1:**
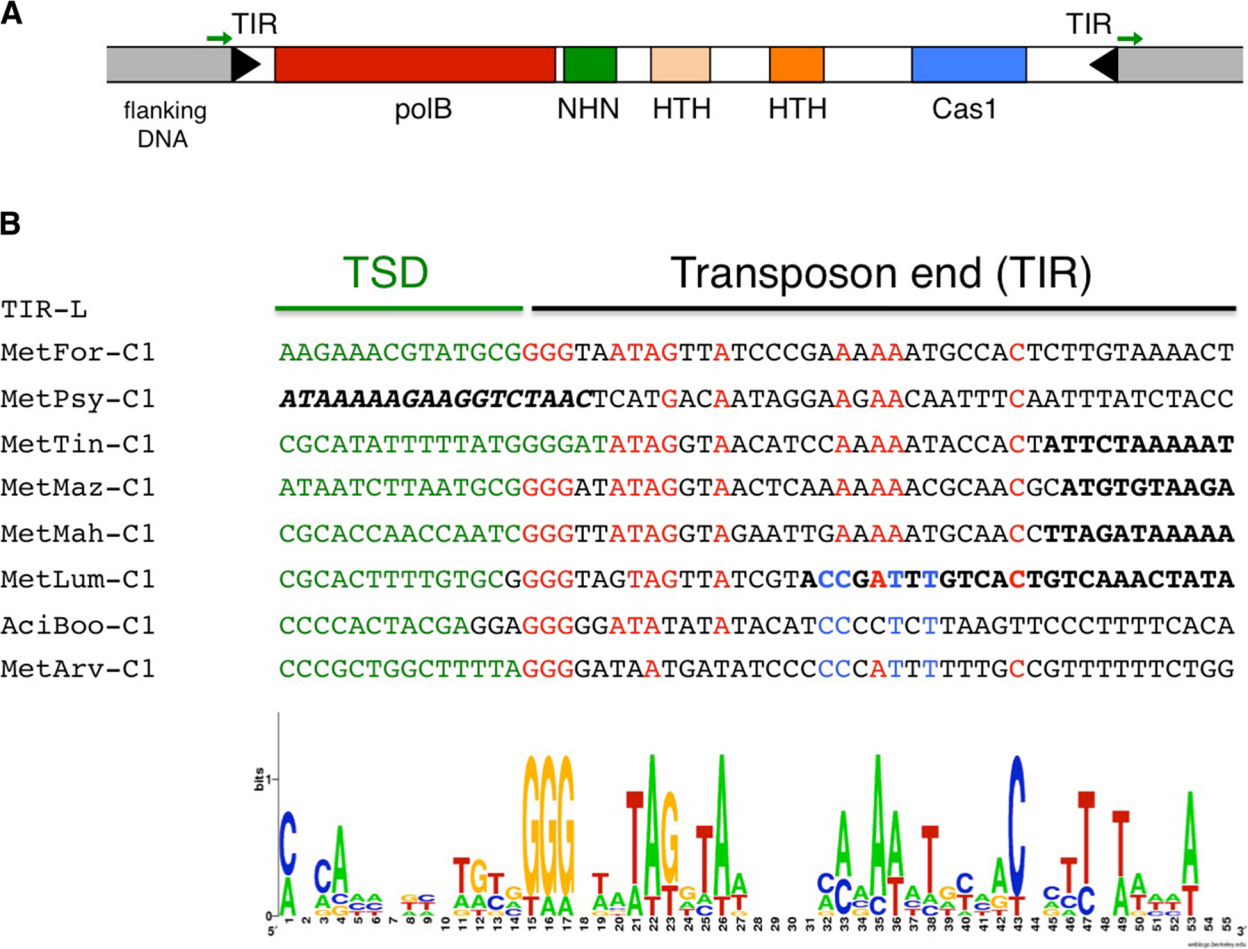
**Properties of the family 2 casposons. (A)** Predicted common protein-coding genes within family 2 casposons include a PolB family polymerase, an HNH family endonuclease, several HTH domains, and Cas1. The gene color code corresponds to that of Krupovic *et al.* The green arrows flanking the casposons indicate target site duplications (TSDs). **(B)** An alignment of the first 41 nucleotides (nt) of casposon family 2 Left End Terminal Inverted Repeats (TIRs) reveals conserved sequence motifs which could be the basis of transposase recognition. Green letters indicate the TSDs and black letters the TIR sequences identified by Krupovic *et al.*, with apparently conserved patterns highlighted in red or blue. Bold black lettering corresponds to nts that were not included in the analysis of Krupovic *et al.* The aligned sequences and the Accession Number and coordinates for each are: MetFor-C1 [NC_019943;1964105..1964159], MetPsy-C1 [NC_018876;190336..190390], MetTin-C1 [NZ_AZAJ01000001; 3015399..3015453], MetMaz-C1 [NC_003901; 3946587..3946641], MetMah-C1[NC_014002; reverse complement of 1332841..1332895], MetLum-C1 [NZ_CAJE01000015; 159864..159918] AciBoo-C1 [NC_013926; 380309..380363], MetArv-C1 [NC_009464; 2695204..2695258].

The work of Krupovic *et al.* builds on a previous report on the evolutionary history of Cas1 proteins which identified two groups of Cas1 proteins not associated with CRISPR loci [9]. One of these groups, designated the Cas1-solo group 2, has Cas1 genes in a conserved neighborhood that usually also contains genes for a B family DNA polymerase, an HNH nuclease, and several helix-turn-helix (HTH) domains (Figure 1A). The current analysis reveals that this conserved region is contained between terminal inverted repeats (TIRs) and is flanked by target site duplications (TSDs), hallmarks of DNA transposons encoding RNase H-like transposases (reviewed in [15,16]). Krupovic *et al.* propose that these features suggest that these regions are mobile genetics elements, and that the Cas1 proteins are required for the integration step of transposition. They further propose that the location of this group of proteins within the Cas1 phylogeny indicates that they likely predate the development of CRISPR-Cas systems.

The parallels between the proposed mechanism of the adaptation step of the CRISPR immune system (reviewed in [17]) and DNA transposition are striking. Cas proteins are responsible for excising a short spacer segment from foreign DNA (typically 32 to 38 bp [11], preceded by a 2 to 5 bp ‘protospacer adjacent motif’, or PAM) and site-specifically integrating it into a particular genomic location at the leader end of a CRISPR locus. Spacer integration is accompanied by the generation of direct repeats on either side of the spacer that can vary in size from 23 to 55 bp [11]. Thus, if the Cas1 nucleases associated with casposons are involved in catalyzing transposition, they presumably can sequence-specifically recognize their TIRs which for most DNA transposons are longer than 10 bp [2,15]. They also appear to exhibit relaxed target DNA recognition properties relative to CRISPR-Cas systems: whereas spacer integration mediated by Cas proteins is site-specific, the genomic locations of casposons suggests that their integration sites are not highly conserved (in line with the integration properties of most RNase H-like DNA transposons with a few notable exceptions, such as the bacterial Tn*7* transposon [18]).

One of the main ways that transposon superfamilies are grouped is by the conservation of TIR sequences located at their transposon ends. At first glance, the 19 putative casposon TIR sequences identified and analyzed by Krupovic *et al*. appear disconcertingly variable both in length and in sequence. However, we find that it is possible to align the TIRs of the sequences corresponding to casposon family 2 members (the most populous casposon family defined in Krupovic *et al.*) such that a pattern of conserved base pairs emerges within the terminal approximately 20 bp (Figure 1B). This suggests that transposon-specific end recognition by a casposon-encoded protein is reasonable. (Casposon families 1 and 3 TIRs can also be aligned to reveal conserved TIR motifs but have fewer representatives than family 2.)

The alignment in Figure 1 also suggests a resolution of a second unusual feature of the sequences presented by Krupovic *et al.*, which is that the TSDs are reported to vary in size from 1 to 27 nucleotides (nt). TSD size is typically highly conserved in Insertion Sequences and DNA transposon superfamilies, rarely varying by more than one or two nt [15,2]. This is because TSD size is a direct consequence of the spacing of the staggered cuts generated by a transpososome assembled on target DNA, and it reflects properties of the distinct architecture - in particular the distance between and the orientation of two catalytic sites - of these multimeric protein-DNA complexes. When the TIRs of casposon family 2 are aligned as in Figure 1B, the TSD size (as they are usually defined which does not include any overlap with the TIRs) now converges on 14 bp. This is relatively large when compared to TSDs of most characterized transposons, but is substantially less than the range of 23 to 55 nt for the repeat size of CRISPR systems. The thus-aligned TSD sequences also hint at yet another feature of many characterized DNA transposons which is a preferred palindromic target site motif [19].

Finally, it should be noted that all of the casposon-associated Cas1 proteins identified by Krupovic *et al.* possess the four conserved catalytic residues expected for an active Cas1 nuclease (Supplemental Figure 1 in their report).

## Conclusions

The evidence is compelling that casposons possess some of the expected properties of active DNA transposons. However, as we are only beginning to understand how the multiple Cas proteins in different CRISPR systems mediate immunity, the evolutionary link between the CRISPR-associated Cas1 proteins and the casposon-associated Cas1 proteins provides only limited insight into the possible mechanism of casposon mobility. Many intriguing questions have been raised by the report of Krupovic *et al.* Since two types of nuclease are often associated with casposons, the Cas1 proteins and usually an HNH nuclease, does the latter have a role? If so, do these nucleases work together and interdependently to catalyze excision and integration? How might Cas1 and a B family polymerase collaborate to generate the proposed intermediate of the reaction, an excised transposon flanked by double-strand breaks? How is this related to the transposition mechanism of the superfamily of self-synthesizing Polinton/Mavericks found in eukaryotes [20,21], to which casposons are proposed to be mechanistically related albeit not evolutionarily [13]? Do the recurrent HTH domains identified within casposons (for example, all the Cas1 proteins of casposon family 2 have a conserved HTH appended to their C-termini) play a role in the recognition of transposon ends or a target site? Clearly, experimental biochemistry is needed to answer these questions.

## Abbreviations

bp: base pair; Cas: CRISPR-associated; CRISPR: Clustered Regularly Interspaced Short Palindromic Repeats; HTH: helix-turn-helix; nt: nucleotides; PAM: protospacer adjacent motif; TIR: terminal inverted repeat; TSD: target site duplication.

## Competing interests

The authors declare that they have no competing interests.

## Authors’ contributions

ABH and FD wrote the text, and approved the final manuscript.
